# Aging compromises human islet beta cell function and identity by decreasing transcription factor activity and inducing ER stress

**DOI:** 10.1126/sciadv.abo3932

**Published:** 2022-10-05

**Authors:** Shristi Shrestha, Galina Erikson, James Lyon, Aliya F. Spigelman, Austin Bautista, Jocelyn E. Manning Fox, Cristiane dos Santos, Maxim Shokhirev, Jean-Philippe Cartailler, Martin W. Hetzer, Patrick E. MacDonald, Rafael Arrojo e Drigo

**Affiliations:** ^1^ Creative Data Solutions, Vanderbilt Center for Stem Cell Biology, Nashville, TN 37232, USA.; ^2^ Integrative Genomics and Bioinformatics Core, Salk Institute of Biological Studies, La Jolla, CA 92037, USA.; ^3^ Department of Pharmacology and Alberta Diabetes Institute, University of Alberta, Edmonton, Alberta T6G2E1, Canada.; ^4^ Department of Molecular Physiology and Biophysics, Vanderbilt University, Nashville, TN 37232, USA.; ^5^ Molecular and Cell Biology Laboratory, Salk Institute of Biological Studies, La Jolla, CA 92037, USA.

## Abstract

Pancreatic islet beta cells are essential for maintaining glucose homeostasis. To understand the impact of aging on beta cells, we performed meta-analysis of single-cell RNA sequencing datasets, transcription factor (TF) regulon analysis, high-resolution confocal microscopy, and measured insulin secretion from nondiabetic donors spanning most of the human life span. This revealed the range of molecular and functional changes that occur during beta cell aging, including the transcriptional deregulation that associates with cellular immaturity and reorganization of beta cell TF networks, increased gene transcription rates, and reduced glucose-stimulated insulin release. These alterations associate with activation of endoplasmic reticulum (ER) stress and autophagy pathways. We propose that a chronic state of ER stress undermines old beta cell structure function to increase the risk of beta cell failure and type 2 diabetes onset as humans age.

## Teaser

As human beta cells age, they become less functional and accumulate cellular stress due to loss of transcriptional control.

## INTRODUCTION

Aging is linked to functional deterioration of proteins, organelles, cells, and tissues, which results in neurodegenerative, cardiovascular, and metabolic diseases (*1*–*3*). For example, type 2 diabetes (T2D) is a metabolic disease caused by the functional decline and/or loss of peripheral insulin signaling, ultimately resulting from the inability of pancreatic beta cells to secrete enough insulin to maintain glucose homeostasis (*4*, *5*). Insulin secretion is controlled by rising glucose levels and by factors released from islet glucagon–secreting alpha cells, islet somatostatin–secreting delta cells, the autonomic nervous system, and the vasculature (*6*–*9*).

Islet beta cells are remarkably long-lived, and many remain postmitotic throughout their lifetime (*10*–*12*). Consequently, these largely postmitotic cells normally maintain their functional integrity for many decades. However, beta cell aging is associated with a higher incidence of T2D and impaired beta cell activity (*5*). Older beta cells have several unique phenotypes, including reduced proliferation rates, increased transcriptional noise, elevated lipid droplet accumulation, senescence, distinct chromatin states, and impaired intracellular calcium homeostasis (*13*–*17*). Accordingly, these phenotypes are expected to undermine beta cell function to impair insulin secretion [reviewed in (*18*)]. Therefore, a better understanding of the underlying molecular mechanisms regulating beta cell structure function across the human life span is needed to better understand how aging affects beta cells and contributes to T2D onset in old age.

Islets are composed of several functionally and transcriptionally discrete beta cell populations, which can secrete insulin in response to glucose stimulation differently and whose relative composition is affected by T2D (*17*, *19*–*21*). Such cellular heterogeneity is driven by distinct transcriptional states that are proposed to involve varying expression levels of beta cell marker genes such as *INS*, retinol binding protein 4 (*RBP4*), or the cellular stress response (*22*–*24*).

In this study, we have combined single-cell transcriptomics, high-resolution tissue confocal microscopy, and functional analyses to reveal the dynamics of the beta cell aging process. Aging beta cells display increased transcriptional output and protein translation loads, up-regulation of the endoplasmic reticulum (ER) stress response, and autophagy, which associates with compromised expression of key beta cell markers and secretory function. Moreover, we reconstructed the architecture of gene regulatory networks (GRNs) formed by human beta cell transcription factors (TFs) and found that GRNs required for proper response to stress are down-regulated in aging beta cells. Our data determine how the molecular heterogeneity and functional profile of human beta cells are modulated over time and identify increased ER stress and autophagy as signatures of beta cell aging.

## RESULTS

### Aging human beta cells have compromised transcriptional identity and secretory function

Beta cell identity can be defined by heterogeneous gene and protein expression levels of beta cells (e.g., *INS*, *RBP4*, *PDX1*, and *MAFA*) and/or cell surface markers (*CD9* and *ST8SIA1*), which identify beta cell populations with different insulin secretion profiles (*16*, *20*, *23*, *25*, *26*). Accordingly, changes to beta cell gene expression, including transcriptional noise (*17*), senescence (*27*, *28*), and compromised identity, are linked to impaired beta cell function during aging and/or T2D pathophysiology (*24*, *25*, *29*).

**Fig. 1.**
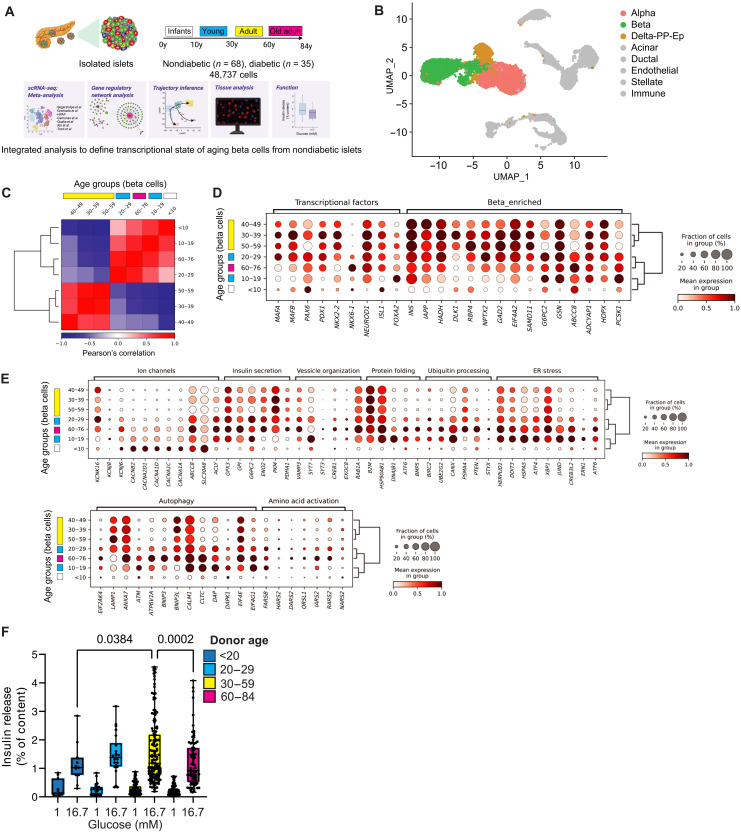
Gene transcription landscape of aging human beta cells. (**A**) Integration of human islet single-cell RNA sequencing (scRNA-seq) and analysis for cell type identity, pseudotime trajectory, TF activity, and GRN analysis. Glucose-stimulated insulin release assays (GSIS) from isolated human islets quantified beta cell function, and confocal microscopy quantified human beta cell TF expression in situ. (**B**) Uniform Manifold Approximation and Projection (UMAP) of our integrated scRNA-seq dataset with 68 nondiabetic (ND) donors, 35 diseased donors, and 48,737 cells in total. Major islet cell types are shown; non-endocrine cells are in gray. (**C**) Pearson correlation matrix and hierarchical clustering analysis (HCA) of human beta cell transcriptomes shows high similarity between beta cells from younger (0 to 29 years old) and older (>60 years old) samples, or between middle age samples (30 to 59 years old). (**D**) Dot plot with HCA of beta cell TFs and beta cell–enriched genes in each decade of life. (**E**) Dot plot with HCA of beta cell ion channel, insulin secretion, vesicle organization, protein homeostasis, stress, autophagy, and amino acid activation genes. (**F**) GSIS of isolated islets from 268 ND donors incubated with 1 or 16.7 mM glucose concentrations and split across different age ranges. Data are shown as a fraction of the total beta cell insulin content. Each point represents one individual donor. Statically significant differences were determined by one-way analysis of variance (ANOVA) with Tukey multiple comparisons posttest.

We hypothesized that aging leads to changes in the beta cell transcriptional identity and compromises beta cell structure function. To test our hypothesis, we first performed a meta-analysis of published single-cell RNA sequencing (scRNA-seq) data of human pancreatic islets (Fig. 1, A and B, and table S1). We used Seurat (*30*) to generate an integrated scRNA-seq dataset with *n* = 68 nondiabetic (ND) and *n* = 35 T2D donors with ages ranging between birth and 76 years of age (Fig. 1A, fig. S1A, and table S1). All major pancreatic cell types were identified on the basis of the expression of well-established gene markers, including beta cells (Fig. 1B; fig. S1, B to D; and table S2), and their relative frequency across donors was very heterogeneous (fig. S1E). Next, we focused on ND beta cells and divided the cells in age groups representing most decades of the human life span and calculated the overall degree of similarity between beta cells in each decade of life (Fig. 1C). We identified two distinct beta cell clusters, one with mostly younger (<30 years old) and older samples (>60 years old) and another with middle-aged adult samples (between 30 and 59 years old) (Fig. 1C). Observation of these clusters was independent of study origin (fig. S1F).

When compared to middle-aged beta cells, younger and older beta cells have lower expression levels of several beta cell identity markers and higher expression levels of genes associated with ion channels, immune response, protein folding and degradation, response to ER stress, autophagy, amino acid activation, incretin signaling, and fatty acid metabolism [as expected, (*13*)] (Fig. 1, D and E, and fig. S2, A and B). This phenotype correlated with changes in the total gene transcription output of beta cells at different decades of life and was observed in male and female donors (fig. S2A). Beta cell gene transcription gradually increases during the first 3 decades of life and remains steady for ~30 years before declining substantially in old age (fig. S2C). A fraction of all old beta cells (~20 to 40%) expressed higher levels of beta cell aging and senescence markers *p21/CDKN1A* and *IGF1R* (fig. S2, A and B) (*28*, *31*), and of the incretin receptors *GLP1R* and *GIPR* (fig. S2B). No age-dependent changes were observed in the other beta cell senescence gene *p16/CDKN2A* (fig. S2A) (*31*, *32*).

We hypothesized that the gradual age-dependent loss of transcriptional maturity of old beta cells would correlate with loss of insulin secretion. We analyzed data from glucose-stimulated insulin secretion assays performed on *n* = 268 ND human islet isolations spanning 8 decades of life, from infants to adults (Fig. 1E and table S3A). We divided the data according to the transcriptional similarity identified in beta cells at different decades of life (Fig. 1C); as expected, we observed a gradual increase in beta cell glucose-stimulated insulin release that peaked between the 3rd and 5th decades of life before declining significantly after the 6th decade of life to levels comparable to young donors (Fig. 1F). No differences in islet insulin content were observed between donor age groups (table S3B).

These experiments establish the temporal dynamics of human beta cell gene transcription at different postnatal stages of the human life span. These data reveal that beta cell aging is associated with reduced expression of several genes important for mature beta cell structure function (despite increased overall transcriptional output), up-regulation of protein homeostasis, immune response, lipid metabolism, and stress genes. This transcriptional architecture associates with impaired secretory function of aging ND beta cells in vitro.

### Aging beta cells have decreased beta cell TF expression and active ER stress response in situ

**Fig. 2.**
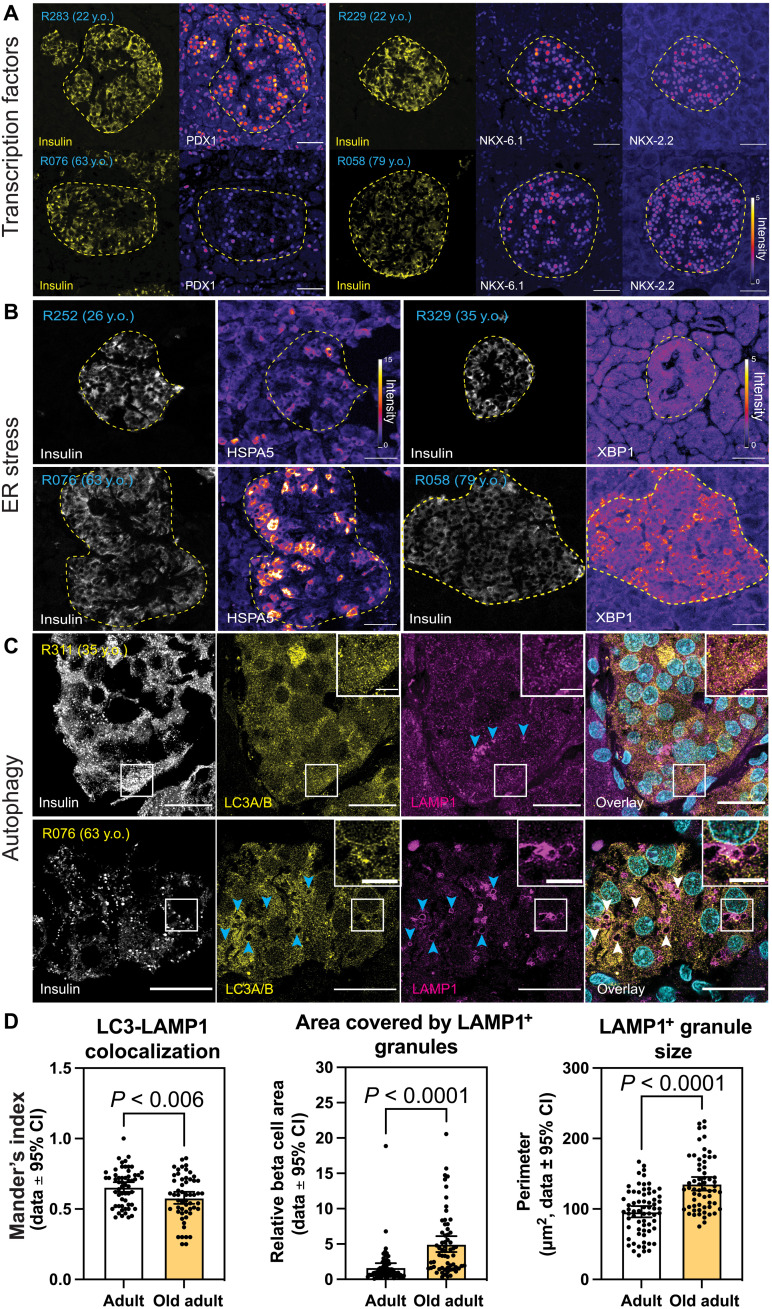
Beta cell TFs, ER stress, and autophagy markers in aging beta cells. Immunohistochemistry and confocal microscopy of human pancreas formalin-fixed paraffin-embedded samples from adults (22 to 35 years old) and old adults (61 to 79 years old). Human islets were stained with (**A**) insulin, PDX1, NKX6-1, and/or NKX2-2; (**B**) insulin and HSPA5 or XBP1; and (**C**) insulin, LC3A/B, and LAMP1. (**D**) Quantification of LC3-LAMP1 colocalization, area of beta cell cytosol covered by LAMP1^+^ granules, and LAMP1^+^ granule size. Each dot represents data from a single region of ~140 μm^2^ of human islets. Here, 1 region per islet, 10 regions per donor were quantified. In (A) and (B), the islet region is demarcated by the dotted yellow line. Scale bars, 50 μm (A and B), 20 μm (C), and 5 μm (inset of C). CI, confidence interval.

Our gene expression analysis indicated that aging beta cells have reduced expression of key beta cell TFs, active ER stress, and autophagy pathways. To validate these findings, we performed immunohistochemistry (IHC) and confocal microscopy of beta cells from ND donors (age range, 22 to 79 years old; see Methods). Here, human pancreas stained with antibodies against INS, PDX1, NKX6-1, and NKX2-2 revealed significant decreases in the nuclear protein levels of all three TFs in old beta cells (20 to 50% versus middle-aged adult controls; Fig. 2A and fig. S2D).

The ER stress response is an adaptive cellular process triggered during times of increased demands for protein folding, and that involves the activation of three interconnected signaling branches, namely, the PERK-eIF2a-ATF4, ATF6, and IRE1 pathways. Together, these pathways enable stressed cells to maintain homeostasis by increasing the expression of chaperone proteins, lipid synthesis, and protein degradation pathways (*33*). Accordingly, genes linked to all three ER stress branches are up-regulated in old beta cells, including their downstream targets such as *HSPA5*, and the ER stress TF *XBP1* (Fig. 1E). To validate these results in situ, we again applied IHC and confocal microscopy of ND beta cells. As expected, we observed increases in cytoplasmic HSPA5 (~13%) and in nuclear and cytosolic levels of XBP1 (23 and 36%, respectively) in beta cells from old ND donors (Fig. 2A and fig. S2D).

We hypothesized that the ER stress in old beta cells was likely due to higher protein synthesis loads, which are expected to arise because of increased transcriptional output and higher mRNA processing in the nucleolus. Notably, increases in nucleolar size correlate with higher rates of protein synthesis in human cells (*34*). We performed confocal microscopy of ND young and old adult beta cells immunostained for the nucleolar marker Nucleolin (NCL), which revealed that old beta cells have significantly larger nucleoli (fig. S2E). Since replication of human beta cells is an extremely rare event during old age (*11*), and increased body weight in adult ND humans does not correlate with higher beta cell mass (*35*), these results are unlikely to be explained by alterations in beta cell replication and regulation of NCL during the cell cycle (*36*). Therefore, these results support our findings of increased transcriptional output in old beta cells and further suggest that mRNA processing and protein synthesis could be increased in these cells.

High rates of protein synthesis are expected to increase the demand for amino acid influx and metabolism and potentially lead to beta cell hypertrophy. First, we analyzed the expression of genes involved in amino acid transport, activation, and biosynthesis. While no age-dependent changes in amino acid biosynthesis genes were observed, old beta cells have increased expression of several amino acid transporters, amino acid activation, and amino acid starvation response genes (Fig. 1E and fig. S3A). Together, this suggests that normal amino acid homeostasis is impaired old beta cells. Unexpectedly, confocal microscopy of ND adult beta cells using the membrane marker E-cadherin revealed that old beta cells are ~10% larger than younger adult beta cells (fig. S3B), which suggests that aging beta cells increase their cell mass despite having compromised amino acid metabolism and homeostasis pathways.

When cells are challenged with amino acid deficits, they up-regulate autophagy to sustain cellular amino acid availability and maintain cell homeostasis. This response is mediated (at least in part) by the suppression of mammalian target of rapamycin (mTOR) signaling (*37*). Accordingly, aging beta cells have high expression levels of several autophagy-associated genes and down-regulation of mTOR signaling genes (Fig. 1E and fig. S3A). We validated these results using confocal microscopy of ND beta cells immunostained with LC3A/B (autophagosome marker) and LAMP1 (lysosome marker), or the mTOR target phospho-S6 kinase (pS6K) (*37*). We found that old beta cells have overall lower levels of cytoplasmic pS6K (fig. S3C), supporting findings of mild down-regulation of some mTOR pathway genes (fig. S2E). Next, we measured the colocalization of LC3 and LAMP1-positive vesicles to access the functional state of the beta cell autophagy pathway, as recently shown in T1D beta cells (*38*). Like T1D beta cells, old ND beta cells have lower LC3-LAMP1 colocalization indexes, which indicate impaired degradation of autophagy vesicles (Fig. 2, C and D). This is associated with accumulation of LAMP1-positive granules in the aging beta cell cytosol (Fig. 2, C and D). Notably, LAMP1 marks lipofuscin bodies, which contain partially digested molecules processed in the lysosomal system (*39*), that accumulate as human beta cells age (*10*) and are associated with compromised brain cell function and health (*39*, *40*). Together, these results support our bulk RNA-seq analysis to reveal that aging beta cells have compromised expression of PDX1, NKX6-1, and NKX2-2, higher gene transcription, ER stress, altered amino acid homeostasis, and autophagy, the latter being impaired and likely explains the age-dependent accumulation of lipofuscin bodies in old beta cells.

### Age-dependent modulation of beta cell transcriptional heterogeneity

**Fig. 3.**
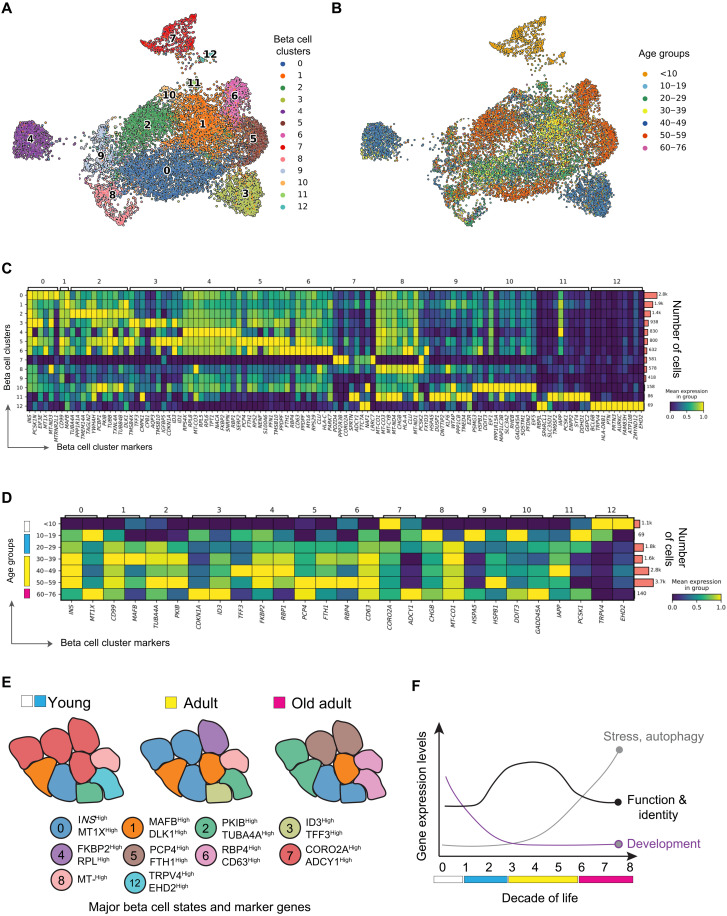
Effect of aging on the prevalence of distinct beta cell transcriptional states. (**A**) UMAP of 11,279 human ND beta cells and transcriptionally different human beta cells. (**B**) Same as in (A); however, each beta cell is colored by the age decade of their respective donor. (**C**) Heatmap of differentially expressed genes per cluster. Rows on the left and top columns provide the identity of each beta cell cluster. Bars on the right show the total number of cells in each cluster. (**D**) Heatmap of differentially expressed genes in different decades of life. Rows on the left indicate decades of age, the top column identifies each beta cell cluster, the bottom column identifies differentially expressed genes found in each cluster, and bars on the right show the total number of cells in each age decade of life. (**E**) Illustration of changes in beta cell state composition associated with transcriptional heterogeneity phenotypes identified from our clustering approach. (**F**) Illustration highlighting the effect of aging on the expression dynamics of beta cell development, stress, function, and identity genes. In (A), cell colors match the colors assigned to each beta cell cluster from (A).

To determine how aging affects beta cell transcriptional heterogeneity, we performed graph-based unsupervised clustering of ND beta cells (fig. S4, A and B) (*30*) and identified 13 transcriptionally distinct beta cell clusters distributed across the human life span (Fig. 3A and fig. S4C). Here, ~75% of infant (<10 years old) beta cells were in clusters 1 and 7 (Fig. 3, A and B, and fig. S4C), while most beta cells from largely young (10 to 29 years old), middle-aged, and older adults (30 to 76 years old) were in clusters 0, 1, and 2 (Fig. 3, A and B, and fig. S4C). Smaller clusters of beta cells were observed in specific age groups (e.g., cluster 3 for 30 to 49 years old and clusters 5 and 6 for 50 to 76 years old) (Fig. 3, A and B, and fig. S4C). Most clusters contained donors from both sexes and from multiple studies, except cluster 4, and therefore will not be analyzed further (fig. S4D).

Differential gene expression for each beta cell cluster identified cluster marker genes; the infant beta cell–rich cluster 7 was characterized by high expression levels of *CORO2A* and *ADCY1* (*27*) and lower levels of *INS* and *MAFB* (Fig. 3C). CORO2A and ADCY1 mark immature stem cell–derived beta cells that lack *NEUROD1* (*41*), thus indicating the relative transcriptional immaturity of cells in this cluster. Remaining infant beta cells were in clusters predominantly occupied by adult beta cells (clusters 0 to 2; fig. S4C), which suggests that some infant beta cells exist in an adult-like transcriptional state marked by high expression of *INS* and *MAFB* (*25*)*, CD99* (*42*), *DLK1* (*15*), and *MT1X* (*43*) (Fig. 3C). In contrast, older adult donor beta cells were mostly in clusters 2, 5, and 6—the latter two being virtually unique to older donors (fig. S4C). Cells in these clusters have higher expression of the protein kinase A inhibitor beta (*PKIB*) and tubulin *TUBA4A* (cluster 2), the calmodulin-binding protein *PCP4,* the beta cell senescence marker p21*CDKN1A* (cluster 5), or the beta cell marker *RBP4* and the lysosomal marker *CD63* (cluster 6) (Fig. 3C). High expression of these genes is linked to immature and/or dysfunctional beta cells (*24*, *44*–*47*), whereas old beta cells have increased autophagy in situ (Fig. 2, C and D). Significant expression of the beta cell heterogeneity marker *ID1* (*26*) was detected in cluster 5; however, expression of beta cell heterogeneity markers *CD9* and *ST8SA1* (*20*) was not exclusive to beta cells and/or expressed at very low levels (not shown).

To test whether aging affects the expression of genes in beta cells within the same cluster, we performed differential gene expression analysis in each isolated cluster and compared cells grouped by decade of age (Fig. 3C). This revealed that beta cells from middle-aged donors (30 to 59 years old) tend to have higher expression of cluster marker genes than infant or older donors (Fig. 3D). Aging beta cells (from donors 50 years old and older) have high expression of beta cell dysfunction and/or immaturity genes (*RBP4*, *PKIB*, *TUBA4A*, *PCP4*, *ADCY1*, and *CDKN1A)*, higher transcriptional output, and lower expression of adult beta cell markers (*INS* and *MAFB*) (clusters 3, 5, and 6; Fig. 3D and fig. S4F).

Pathway enrichment analysis revealed that clusters with most adult and old beta cells (0, 1, 3, 5, and 6) are overrepresented by oxidative phosphorylation, protein synthesis, and secretory pathways (fig. S4D). Clusters with mostly infant beta cells were enriched for cell morphogenesis and differentiation pathways (fig. S4E); a fraction of old beta cells (~25%) were in cluster 1 (fig. S3C), which is enriched for protein transport, autophagy, inflammation, protein degradation, and stress pathways (fig. S4E). These findings support that a significant fraction of old beta cells have increased transcriptional output, activated cell stress, and autophagy pathways (Figs. 1 and 2 and fig. S4E).

Next, we tested whether aging of ND human beta cells accumulates DNA damage, which has been associated with beta cell senescence (*27*). We monitored beta cell DNA damage using confocal microscopy of human pancreas formalin-fixed paraffin-embedded (FFPE) sections immunostained for the tumor suppressor p53-binding protein 1 (53BP1), which accumulates at sites of double-strand breaks to form nuclear foci (*48*), or the nucleic acid oxidative damage reporter 8-hydroxy-guanosine (8HG). As expected, nucleoplasm beta cell 53BP1 levels are higher than in surrounding cells (*27*). However, rare 53BP1-positive foci in beta cells were found independently of donor age (fig. S4G and table S8). 8HG levels were slightly increased in the nuclear and cytosolic compartments of older beta cells (3 and 7%, respectively; fig. S4H).

These results reveal that the transcriptional heterogeneity of human beta cells is modulated over long periods of time (Fig. 3, E and F). This phenotype consists of the reorganization of beta cell subpopulations in aging donors, where beta cells express genes linked to beta cell dysfunction, stress, senescence, and autophagy. This transcriptional architecture likely supports increases in ER stress, autophagy, DNA damage, and senescence processes in situ that undermine normal beta cell secretory function (Figs. 1 and 2 and figs. S2 to S4) (*27*, *31*).

### Identity of GRNs underlying beta cell transcriptional heterogeneity

Development and maturation of beta cells depend on the coordinated activity of several TFs (e.g., PDX1, MAFA, and NKX6-1) that target sets of genes to form GRNs that maintain normal beta cell identity and function (*49*). Decreased beta cell TFs levels are observed in T2D and underlie compromised beta cell function and identity (*29*). However, it remains largely unknown how beta cell TF GRNs are affected during development and aging.

**Fig. 4.**
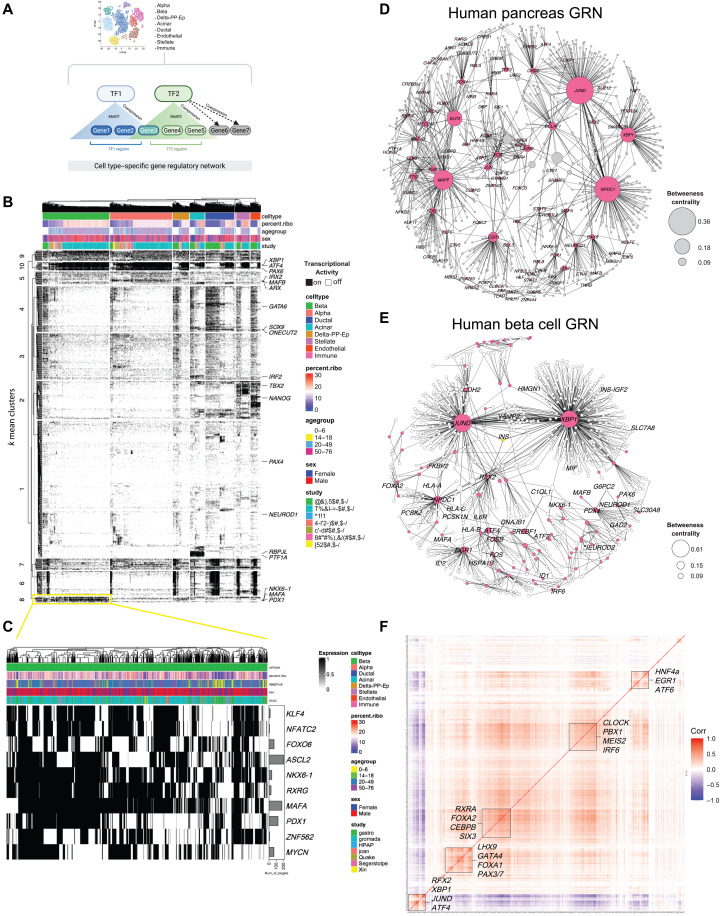
Identification of beta cell GRNs. (**A**) Illustration of SCENIC analysis. (**B**) Heatmap and hierarchical clustering analysis (HCA) of TF activity patterns. TFs classified as “ON” are shown in black, while TFs classified as “OFF” are in white. Top rows show the distribution of cells within the heatmap subdivided by cell type, age group, percent of ribosomal genes, sex, and study origin. Column dendrogram represents HCA clades within each cell type using “Euclidean” distances. Rows are TFs identified using SCENIC. Beta cell–enriched TFs are in (**C**). (**D** and **E**) GRNs formed by TFs identified using SCENIC in the human pancreas and in beta cells, respectively. TFs are shown as pink nodes, while target protein-coding genes are shown in light gray in (D) and white in (E). Black lines connecting the nodes represent the importance metric (IM) between TF-gene pairs, where thicker edges indicate stronger TF-target relationship. Node size represents the “betweenness centrality” measurements that report on the influence of a given TF within the network. (**F**) Pearson correlation matrix of 609 human beta cell TFs. Correlation index scale is on the right. Bounding boxes highlight clusters of TFs with high degree of correlation.

To answer these questions, we applied pySCENIC to infer TF activity and create TF-specific GRNs (*50*, *51*). pySCENIC determines gene coexpression networks for each cell based on scRNA-seq data, where the TF-gene relationship is evaluated using cis-regulatory TF database containing *n* = 1839 TFs and their consensus motifs (Fig. 4A). Each TF-gene set pair with significant motif enrichment is included within a TF-specific regulon that is scored and transformed into a binary matrix that reports on TF activation in each cell (0 = TF “OFF” and 1 = TF “ON”). This binary matrix is scored using GRNBoost2 to create a TF importance metric (IM) based on the frequency of ON or OFF events for each TF identified. Here, only regulons with the highest IM scores (>85% percentile, to select very strong TF-gene relationships) were used to reconstruct cell type–specific GRNs (Fig. 4A and table S5).

We applied pySCENIC to our integrated ND human pancreas scRNA-seq dataset to identify cell type–specific regulons (Fig. 4B). As expected, we identified *PAX6* as regulon of all endocrine cells; *PDX1*, *NKX6-1*, *MAFB*, and *MAFA* in beta cells, *IRX2* and *ARX* in alpha cells, *PTF1A* and *NR5A2* in acinar cells, and *SOX9* in ductal cells (Fig. 4, B and C, and fig. S5, A and B). *XBP1*, *JUND*, *NPDC1*, and *ELF3* were identified as major regulons across all cell types (Fig. 4B and fig. S5B). To visualize the relative importance of individual TF regulons, we generated GRN graphs with TF-gene pairs (Fig. 4D). As expected, *XBP-1*, *JUND*, *MAFF*, *NPDC1*, and *ELF3* formed large interconnected nodes to create “TF hubs,” while cell type–specific regulons have less influence in the GRN organization (Fig. 3D and fig. S5A).

In beta cells, we identified *n* = 609 TFs and their regulons (Fig. 4E). *PDX1* and *NKX6-1* formed an interconnected network with other beta cell TFs (*PAX6*, *MAFB*, and *NEUROD1*) (Fig. 4E). Beta cell TFs targeted up to 200 different genes, including beta cell function and identity genes (*SLC30A8*, *GAD2*, and *G6PC2*) (Fig. 4, C and E). *JUND*, *XBP1*, and *NPDC1* formed regulons connected with beta cell function and identity (*CHGA*, *INS*, and *PCSK1-2*; Fig. 4E), protein synthesis, and/or the ER stress response genes (fig. S5C). Similar analysis revealed the architecture of GRNs created by TFs associated with amino acid starvation and autophagy processes, which are up-regulated in old beta cells (Figs. 1 and 2 and fig. S5, D and E). Of note, the well-established link between *INS* and beta cell TFs (e.g., *PDX1*) was not among the top 15% ranked regulons (Fig. 4E and table S4).

To determine the degree of TF activity coordination in human beta cells, we generated a correlation matrix using the pySCENIC data (Fig. 4F). This revealed two TF groups with mostly anticorrelated activities: (i) a smaller group (*n* = 36) with TFs associated with maintenance of beta cell health via the ER stress response [*CEBPD*, *XBP1*, *ATF4*, and *JUND* (*52*–*55*)] and (ii) a larger TF group (*n* = 573) with *PDX1*, *NEUROD1*, *SIX3*, and the ER stress *ATF6*. Pathway enrichment analysis revealed that the smaller TF group contains cell development and aging TFs, while TFs in the larger group were linked to cell fate commitment, differentiation, and development (fig. S5, F and G). No correlation between key beta cell identity TFs was observed, except between *NEUROD1* and *PDX1* (Fig. 4F and fig. S4H).

Together, these analyses identify the cell type–specific architecture of GRNs in the human pancreas to provide insights regarding the level of TF activity coordination in human beta cells. In these cells, activation of ER stress configures a unique beta cell transcriptional architecture. In contrast, activity of beta cell identity TFs appears to be uncoordinated in relation to most predominant TF activity clusters.

### Development- and aging-specific modulation of human beta cell GRNs

Postnatal development of human beta cells involves the change from a proliferative to a largely postmitotic cell state, which lasts many decades (*10*, *11*). Changes in histone marks allow activation of genes required for adult beta cell structure function, which becomes impaired and dysregulated as humans age (Figs. 1 to 3) (*14*, *15*, *27*, *28*). These changes imply that the activity of beta cell TFs is modulated during development and aging to support age-specific transcriptional signatures.

**Fig. 5.**
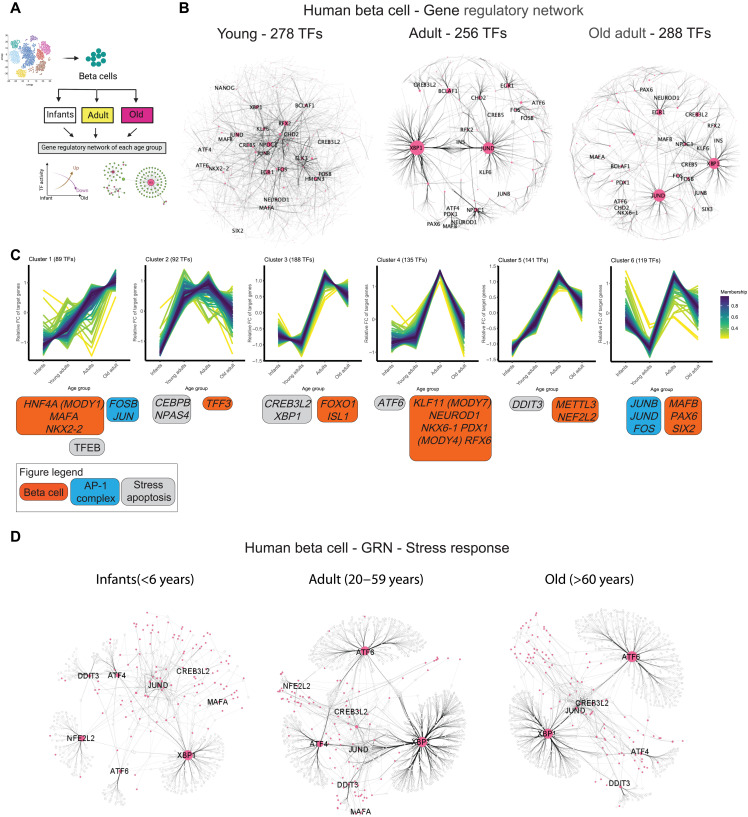
Changes in the TF-GRN landscape of human beta cells in young, adult, and old adult humans. (**A**) Schematics of the approach used to generate age group–specific GRNs. (**B**) Network graphs illustrating the GRNs formed by TFs identified using SCENIC in human beta cells from young (<6 years old), adult (20 to 59 years old), or old adult (>60 years old) donors. TFs are shown as pink nodes, while target protein-coding genes are shown in light gray. Black lines connecting the nodes represent the IM between TF-gene pairs, with thicker edges indicating stronger relation of TF-target. The size of each node represents the betweenness centrality measurements (often used, as a measure of “hub status” due to its influence in flow of information) of a given TF within the network. (**C**) Clustering analysis identified by TCseq resulted in six different families of TFs based on the number of targets gained or lost over increasing age. Identity of select TFs belonging to each family is shown at the bottom of each graph. (**D***)* Same as in (B); however, these GRNs are focused on ER stress–related TFs to highlight age-dependent differences in the organization and scope of ER stress GRNs in human beta cells.

To assess how individual TF regulons are regulated and/or modified during the human life span, we applied pySCENIC to beta cells categorized according to their age and transcriptional phenotype (infants ≤ 10 years old, adult = 20 to 59 years old, old adult > 60 years old; Figs. 1 and 5A). This identified up to *n* = 288 TFs in each age group (Fig. 5B). The GRN of infant beta cells contained small TF nodes, which could explain the reduced transcriptional output of these cells (Fig. 5B and fig. S2C). In contrast, old adult beta cell GRNs are centralized to *XBP1* and *JUND*, whereas beta cell TFs (*PAX6* and *PDX1*) have a limited contribution to the overall GRN organization across age groups, where old beta cells have ~10% more TF nodes than younger adult beta cells (Fig. 5B). In addition, old beta cell GRN includes activation of *DDIT3* (also known as *CHOP*) and *ATF6* (Fig. 5B and fig. S6, A and B), which are involved in the beta cell ER stress response (*56*, *57*), and of *CREB3L2*, which is required for increasing the protein translation machinery in secretory cells challenged with high demands for protein translation (fig. S5, A and B) (*58*).

Next, we hypothesized that human beta cell aging would be associated with the reconfiguration of the beta cell GRN architecture, including changes to TF regulons involved in the ER stress response. To test this hypothesis, we combined the pySCENIC data from infants (*n* = 255 TFs), adults (*n* = 278 TFs), and old adults (*n* = 288 TFs) to generate a unique list of TFs (*n* = 379). Next, we quantified the total number of genes targeted by each TF (i.e., regulon size) in each age group and performed nearest-neighbor clustering (k-NN) analysis to identify six different clusters of TFs with similar modulatory patterns over time (Fig. 5C). This revealed that most beta cell TFs achieve a maximum regulon size by adulthood and that these TF regulons become significantly smaller during old age, except for TFs in cluster 1 (Fig. 5C). These data illustrate that beta cell aging is associated with GRN reconfiguration and limited TF regulon landscapes. Notably, TFs linked to mature-onset diabetes of the young [*MODY*, *HNF4A* (*MODY1*), *PDX1* (*MODY4*), and *KLF11* (*MODY7*)] gain a significant number of target genes after 2 decades, which could explain how mutations in these genes can affect beta cell structure function to cause diabetes later in life (*59*–*61*).

**Fig. 6.**
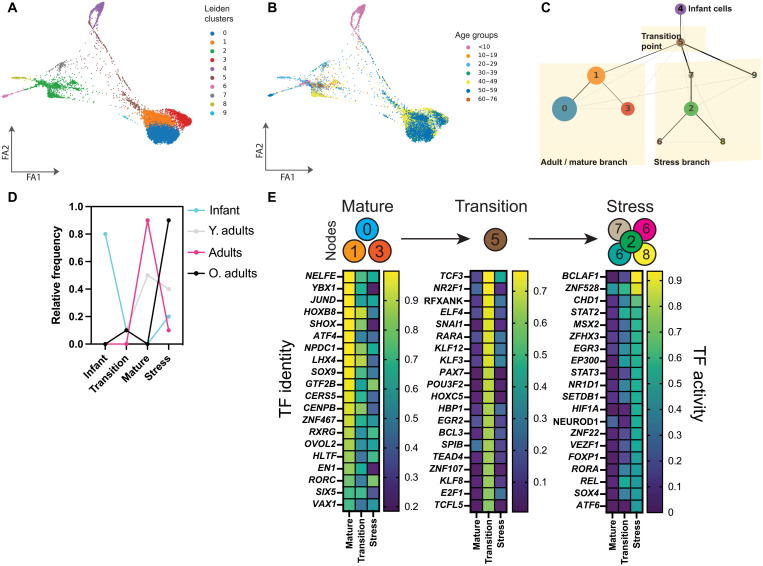
Pseudotime ordering of human beta cells classified by TF activity at the single-cell level. (**A** and **B**) Force-directed layout to project the Leiden clusters of human beta cells from ND donors analyzed using SCENIC into two dimensions. Distinct Leiden clusters representing distinct beta cell TF states (A) and decades of age for each cell (B) are shown. (**C**) Trajectory analysis performed with partition-based graph abstraction (PAGA) on SCENIC binary output of ND beta cells’ regulons. Trajectory starts at node number 4 (containing mostly infants), and each following node represents a significant point in the trajectory characterized by a distinct TF signature. (**D**) Three pseudotime trajectories are shown, namely, transition, mature, and stress, and their respective cell frequency. (**E**) TF activity identified as differentially modulated across the three different pseudotime trajectories.

This analysis also revealed that old beta cells have small *XBP1* and *ATF6* regulons, thus suggesting that ER stress TFs have a limited and/or different arsenal of target genes. To test this hypothesis, we generated stress response GRNs to determine the contribution of individual ER stress response TFs, their regulon size, and identity of genes targeted in infants, adults, and old adult beta cells. Infant beta cells have small ER stress GRNs that are mostly centered on *XBP1* (Fig. 5D); adult and old adult beta cells have larger GRNs anchored on all three major ER stress TFs (*XBP1*, *ATF4*, and *ATF6*). This approach also revealed that old beta cells have a small *ATF4* regulon with ~75% less genes than adult beta cells (Fig. 5D and fig. S6C). In general, the beta cell *ATF4* regulon contains hundreds of genes involved in ER stress response and protein homeostasis (fig. S6D). However, besides a narrow scope of target genes, the *ATF4* regulon in old beta cells is very different from adult beta cells (fig. S6C and table S6). A similar phenotype was also observed for *XBP1* and *ATF6* regulons, however at different magnitudes (Figs. 5D and 6C and table S6). Adult beta cell *XBP1*, *ATF6*, and *ATF4* regulons target several genes important for beta cell identity and function (*ABCC8* and *ENTPD3*) and ER stress response and/or protein homeostasis (*ATF4/6* and *HERPUD1*) (*52*, *57*). Unexpectedly, these genes are mostly absent from XBP1/ATF4 to ATF6 regulons in old beta cells, which instead can target proapoptotic (*DDIT3*) and senescence (*CDKN1A*) genes (Fig. 5D and fig. S6, D and E).

These results characterize the overall dynamics of human beta cell GRNs to reveal that old beta cells have a modest increase in the number of active TFs (which could contribute to increased transcriptional noise) and limited TF regulon landscapes. This phenotype is associated with reconfiguration of TF regulons required for proper ER stress response that could undermine the efficacy of the ER stress response in old beta cells, and thus contribute toward and/or sustain pathways that negatively affect beta cell structure function as humans age.

### Activation of specific TFs underlies the aging beta cell ER stress phenotype

Next, we sought to determine the identity and TF activity patterns during beta cell aging at the single cell level. We performed pseudotime trajectory inference [i.e., partition-based graph abstraction (PAGA) (*62*)] on binary scoring of all 609 TFs identified in beta cells using pySCENIC. PAGA partitions the cells into “cell nodes” based on expression (in this case, TF “ON”/“OFF”) and tests for links between nodes to construct a graph reflecting a likely “developmental” relationship. Using this approach, we identified several beta cells nodes, each represented by a unique TF regulon signature and age group distribution (Fig. 6, A to C). We set beta cells from the youngest donor (0 year old) as the anchor point for the start of the trajectory, and all other beta cells were distributed in six potential cell trajectories (Fig. 6C and fig. S7A). Since most adult beta cells were in nodes 0, 1, and 3, and most old beta cells were in nodes 2 and 6 to 9, we termed these nodes as “adult” and “stress,” respectively (Fig. 6C). The node between infants and adult/stress nodes was called the “transition zone” and contained a mix of beta cells from all ages (Fig. 6D).

This approach identified TF signatures as beta cells progress from infanthood toward adulthood, and as adult beta cells transition toward stress nodes (fig. S7, A and B). Infant beta cells moving toward the transition zone and adult branches down-regulate *RFX3*, *KLF3*, and *FOXA2*, and up-regulate all major TF regulons associated with adult beta cell GRNs (*XBP1* and *JUND*; Figs. 4 and 5 and fig. S7C). Notably, the pseudotime signature of beta cell identity TFs (e.g., *PDX1*) was heterogeneous (fig. S7B) (*25*, *26*, *28*, *46*), which suggests that beta cell transcriptional heterogeneity and identity are modulated over time by TF ON/OFF patterns.

Next, we investigated the identity of TFs modulated during the transition of adult beta cells toward the stress nodes (path 6; Fig. 6E and fig. S7A). This transition involved the down-regulation of adult beta cell function/health homeostasis (*JUND* and *ATF4*) and postmitotic cell differentiation TFs (*LHX4* and *NPDC1*) (*52*, *55*, *63*, *64*). Adult beta cells in the transition zone transiently activate several TFs associated with early development [such as *KLF* genes, *PAX7*, and retinoic acid receptor alpha (*RARA*)] (Fig. 6E). As expected, beta cells in the stress nodes display strong activation of ER stress TFs (*XBP1*, *ATF6*, and loss of *ATF4*), as well as TFs involved in apoptosis (*BCLAF1*) and hypoxia response (*HIF1A*) (Fig. 6E and fig. S7C). These results identify the TFs modulated during human beta cell postnatal development and/or aging processes and provide a broad overview regarding patterns of TF activity underlying the transition of adult beta cells toward a less mature- and transcriptional-compromised beta cell state(s) that are predominantly found in aging donors.

## DISCUSSION

In the brain, aging cells have compromised nuclear homeostasis, impaired transcriptional control and protein homeostasis, and accumulation of lipofuscin bodies (*1*, *2*, *10*, *40*, *65*). Beta cells are mostly postmitotic cells that can persist throughout the organism’s lifetime (*10*, *12*). Accordingly, we demonstrate that reorganization of beta cell TF composition and GRN signatures in old beta cells associates with increased gene transcription, up-regulation of protein synthesis pathways, ER stress, impaired autophagy and accumulation of lysosomal digestion products, and reduced insulin secretion in vitro. These results are supported by previous studies showing that aging beta cells accumulate lipofuscin bodies (*10*) and are subject to increasing islet fibrosis and inflammation (*66*), senescence (*27*, *28*, *32*), higher transcriptional noise (*17*), and impaired intracellular calcium dynamics (*14*).

**Fig. 7.**
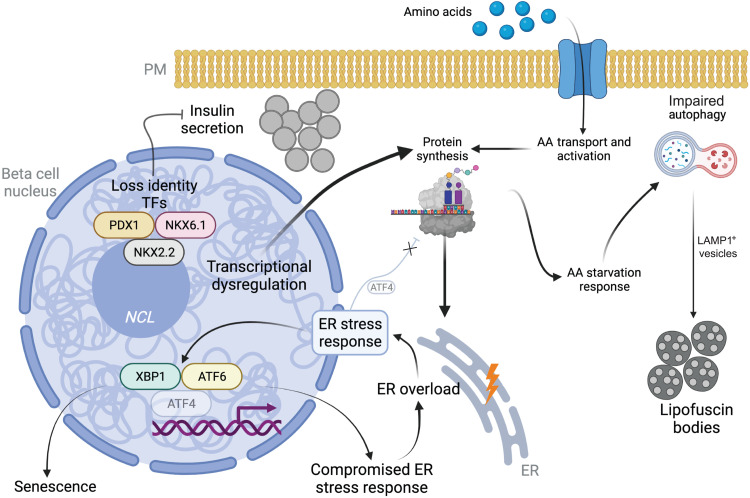
Working model of ER stress activation in old human beta cells. Up-regulation of the beta cell transcriptional output increases protein translation and folding demands. To meet this increased demand, beta cells increase amino acid transport and metabolism. This is also associated with activation of the amino acid starvation response that stimulates autophagy. This process is impaired and leads to accumulation of lipofuscin bodies. Higher protein synthesis overloads the ER and triggers ER stress response pathways, which are compromised. This likely contributes to activation of other beta cell aging–dependent pathways, including senescence. Unresolved ER stress phenotype, combined with compromised expression of identity TFs, creates a chronic ER stress state that undermines beta cell structure function that could lead toward secretory failure and/or death.

We propose that aging causes a rearrangement of GRNs that drives increased beta cell transcriptional output and noise (*17*). This leads to transcriptional and functional immaturity phenotypes, mediated in part by loss of PDX1/NKX6.1/NKX2.2, and drives higher rates of protein synthesis that overload the ER protein folding machinery to trigger (and/or contribute) to ER stress (Fig. 7). Old beta cells have higher expression of the incretin receptor GLP1R, which could represent an adaptive mechanism to preserve beta cell structure function by modulating the ER stress response, including ATF4 (*67*, *68*). However, since the ATF4 GRN is limited in old beta cells (Figs. 5 and 7), these cells are unable to suppress protein translation to alleviate ER stress, which could explain their immature phenotype [Fig. 7, (*52*)]. Consequently, old beta cells up-regulate amino acid transport and metabolism to meet higher amino acid demands to sustain the metabolic demands of the protein synthesis machinery. However, the activation of several amino acid starvation response genes (Fig. 1 and fig. S2) suggests that this adaptive response fails to meet the cell’s amino acid demands. This would explain the up-regulation of autophagy (Figs. 2 and 7), possibly via down-regulation of mTOR signaling (fig. S3). Significant accumulation of LAMP1^+^ vesicles and loss of colocalization with LC3A/B suggest that autophagy is impaired and results in the accumulation of lipofuscin bodies (*10*). Together, these pathways would create and/or sustain a chronic ER stress state in old beta cells that compromises cell function (Fig. 7). *PDX1* regulates beta cell survival under ER stress by interacting with ATF4 (*69*). This raises the possibility that loss of PDX1 in old beta cells may contribute to deficits in ATF4 regulon activity observed during beta cell aging (Fig. 5). Furthermore, decreased expression of key beta cell TFs, up-regulated ER stress, and impaired autophagy are found in T1D and T2D beta cells, which are functionally impaired and/or transcriptionally immature (*29*, *38*, *70*, *71*). Last, induction of chronic ER stress in mouse beta cells accelerates the expression of beta cell aging markers (*28*). Together, these studies underscore the important link between ER stress and normal beta cell structure function and homeostasis in humans. Therefore, approaches that ameliorate and/or modulate beta cell ER stress (e.g., incretins) could be a promising avenue for protecting the function and molecular identity of adult beta cells during old age.

### Limitations of this study

Our results rely on meta-analysis of published scRNA-seq datasets, microscopy, and in vitro experiments. While the scRNA-seq data originate from different laboratories around the globe, likely minimizing center-specific variability introduced by different islet isolation protocols and procedures, this technology has limited potential to detect low-expressing genes and/or rare transcriptional events (*72*). The pySCENIC results are by default constrained by information provided in a curated list of known TFs and their motifs. Therefore, our current analysis and future studies are likely to benefit from new discoveries of TFs and/or motifs. Tissue specimens used for microscopy are from 12 donors processed in a single large isolation center [Alberta Diabetes Institute (ADI), Edmonton, Canada], and the scRNA-seq dataset contains 68 ND donors in total. Therefore, this study may not capture all complexities of beta cell aging in the human population at large. IHC protocols can be inaccurate in quantifying absolute changes in protein expression; to mitigate this, we took advantage of photon-counting spectral detectors that have a wide dynamic range and that were configured to isolate autofluorescence (which can vary depending on lipofuscin body composition, sample fixation, and processing) and high-intensity photons into separate channels to quantify the relative number of photons emitted by specific antibody–fluorophore complexes across age groups. We propose that nucleolar expansion correlates with elevated protein translation in aging beta cells, as observed in other aging cell models (*34*). However, we were unable to directly measure protein synthesis in aging beta cells using our tissue bank samples because of a lack of available technology to quantify protein synthesis rates in situ and/or at the single-cell level. Last, our conclusions are based on data mostly representing western populations to determine predominant transcriptional and functional beta cell phenotypes during aging. Therefore, more experiments are needed to evaluate causality of specific TF activation patterns and the contribution of donor genotype and ancestry to beta cell aging phenotypes.

## METHODS

### Packages and analysis tools

A complete list of software algorithms and package versions used to analyze the data is listed in table S8.

### Analysis of publicly available single-cell RNA-seq data, quality control, and clustering analyses

Raw reads from seven publicly available datasets were obtained as follows: raw data from National Center for Biotechnology Information (NCBI) Gene Expression Omnibus (GEO) accession numbers GSE81547 (*17*) and GSE81608 (*21*) or from ArrayExpress accession E-MTAB-5061 (*26*) were aligned to the hg19 human genome using STAR (STARsolo) version 2.5.3a. Mapping was carried out using default parameters, filtering noncanonical introns, and allowing up to 10 mismatches per read and only keeping uniquely mapped reads. Raw transcript counts were obtained using HT-Seq. Normalization of cell-specific biases was performed using the trimmed mean of M values (TMM) normalization and scaled by the library size. The read counts were log-transformed and size factor–adjusted. We filtered out cells that (i) have a low total normalized counts (bottom 5%) and (ii) have a higher fraction of mitochondrial reads (top 5%). To impute the dropout events and improve the performance of clustering and visualization, we used drImpute. To account for the differences in between the donors and studies, we used Combat. Aligned and preprocessed expression count table data from GSE114297 (*22*) and GSE124742 (*24*) were downloaded directly from NCBI GEO. Data from EGAS00001004653 (*73*) were downloaded as a preprocessed Seurat object. Data from the Human Pancreas Analysis Program (*74*) were downloaded as gene expression count matrices. Subsequent preprocess, filtering, correcting for potential dataset-driven variance(s), and integration were performed by Seurat (v3.2.3) R package (*30*). Individual Seurat objects (per dataset) were filtered to only include genes expressed in three or more cells and exclude low-quality cells/doublets (cells with genes <200 and >8000, mitochondrial percent >25%). Gene expression per dataset was normalized for each cell by library size and log-transformed using a size factor of 10,000 molecules per cell. To further account for doublets of alpha and beta cells, threshold on expression values of insulin (*INS*) and glucagon (*GCG*) were set independently per dataset based on bimodal distribution of *INS^+^* and *GCG^+^* log expression (Unique Molecular Identifier per 10,000 molecules) such that cells coexpressing high levels of two genes were excluded from further analysis. Infants’ cells (age <6 years old) were excluded from this approach since infants’ transcriptome are unique and not the focus of this manuscript. All seven datasets were further merged, normalized, and batch-corrected using the “FindIntegrationAnchors” and “IntegrateData” functions from Seurat package. After integration, corrected data were scaled to unit variance and zero mean, and principal components analysis (PCA) was used for dimension reduction. An elbow plot, which ranks the principal components (PCs) based on percentage variance per PC, was considered to use only 20 PCs for cluster generation downstream. Clustering was performed using the Louvain algorithm implemented in Seurat (resolution = 0.7), and cell types were annotated on the basis of known cell markers (*15*, *16*, *23*, *25*, *26*) and Uniform Manifold Approximation and Projection (UMAP) visuals of marker genes. Subset of ND beta cells (*n* = 11,279 cells) was further scaled to unit variance and zero mean and processed for cluster generation (Fig. 2A) performed using the Louvain algorithm (*30*) on the 20 PCs (resolution = 0.5). Visualization of Figs. 1 (C and E) and 3 (A to D) was done through Scanpy.

### GRN inference

To infer the GRN of healthy pancreatic beta cells, we performed SCENIC (*51*) using pySCENIC functions (*50*). This protocol allows the reconstruction regulons (TF and known target genes) from gene coexpression data, assesses regulon activity in single cells, and can be used to find regulon-enriched cellular clusters. Specifically, we ran v0.11.0 of pySCENIC in a Singularity container built from the Docker Hub image, on ACCRE, Vanderbilt’s High Performance Computing cluster. Following the quality control and feature selection performed in Seurat, we exported its raw counts to a matrix that was then converted to a LOOM file. Alongside a list of 1839 human TFs, this gene expression matrix served as input for calculating gene coexpression modules via GRNBoost2. To account for the stochastic nature of GRNBoost2, we calculated the coexpression modules 100 times and then retained only TF/target gene associations that exist in at least 80% of the runs. We then merged the results of these 100 runs as a left outer join operation and averaged the IM values reported for each association. This consensus GRN was then used as input for module pruning, where we filtered out indirect gene targets lacking the cis-regulatory motif associated with the TF. This step used SCENIC’s RcisTarget and ranking databases for motifs (hg19-500bp-upstream-7species.mc9nr.feather) in the promoter of the genes [up to 500 base pairs (bp) upstream the transcriptional start site (TSS); hg19-500bp-upstream-7species.mc9nr.feather] and 20 kb around the TSS (±10 kbp, hg19-500bp-upstream-7species.mc9nr.feather). Note that we removed 21 TFs with only one motif since we identified at least one gene (*UQCRB*) that was wrongly annotated as a TF. The resulting coexpressed TF-target genes are then grouped into regulons. Last, the activity of the regulons was computed using SCENIC’s AUCell function, which uses the “area under the curve” (AUC) to calculate whether a subset of the input gene set is enriched within the expressed genes for each cell. These activity data were further binarized (assigned an ON or OFF value, per regulon, per cell) by threshold on the AUC values of the given regulon. Both the AUCell and binarized regulon activity matrices were integrated into Seurat object via the “CreateAssayObject” function, for downstream analysis and visualization. Heatmap visuals of binary regulon matrix (Fig. 3) were performed by ComplexHeatmap R package. Network plots were created from Cytoscape from top 15% for ND beta cell subset and top 15% for all cell types based on IM reported for TF/target association from the coexpression modules.

### Regulon coexpression clustering

To identify common patterns of TF activity, we examined the number of downstream target genes across age groups by first compiling a data table containing this information and then calculated ratios of activity using the infant time point as the baseline. We then used the Bioconductor package TCseq to perform *z*-score transformation and clustering analysis of this time course data and identified six distinct patterns of changes of downstream target gene frequencies across age groups.

### Trajectory inference on binary regulon data

To explore cell state transition among various age groups, we inferred global topology of binarized regulon activity matrix (only ND beta cells) yielded from pySCENIC workflow, by applying PAGA. Preprocessing was done in Scanpy, where the binarized regulon matrix was subjected to performing PCA, computing neighborhood graph, and subsequently Leiden clustering (resolution = 0.4) to partition into 12 clusters. Next, PAGA was applied by “scanpy.tl.paga” function implemented in Scanpy, which estimates the connectivity of partitions (Leiden clusters) to depict a graph-like map that assigns continuous transition among Leiden clusters. PAGA results were visualized with Scanpy “draw_graph” function via init_pos=‘paga’ to get embeddings associated to the global topology. Clusters 9, 7, and 4 consisted mostly of age group <6 years, hence were chosen as root cells (starting point cluster) along the PAGA paths. Changes along the PAGA inferred path for marker genes were visualized using “sc.pl.paga_path” function.

### Islet IHC

We obtained FFPE human cadaverous islet sections (table S7) through our in-house human pancreas program (www.isletcore.ca). Samples from University of Alberta do not have RRID (resource research identifiers). IHC of FFPE human pancreas sections was performed as follows: First, the sections were preheated to 60°C for 20 min to soften the wax before immersion in fresh xylene for 16 hours. Next, the slides were rehydrated by 5-minute incubations in a gradient of ethanol, from 100 to 50% (v/v). Slides were washed twice in distilled water and then subjected to heat-induced antigen retrieval in fresh sodium citrate buffer (10 mM sodium citrate, pH 6) and heated using a conventional microwave oven. Samples were cooked until the solution reached a boil and maintained in simmering conditions for 12 min. Next, slides were cooled down to room temperature inside a chemical hood and then incubated for 1 hour with 150 μl of blocking and permeabilizing solution containing 0.3% Triton X-100 and 3% bovine serum albumin (BSA; Sigma-Aldrich). After blocking, the tissue sections were incubated overnight at room temperature with primary antibodies raised against insulin [guinea pig, Dako (catalog no. IR002, lot no. 10149939) at 1:4], 53BP1 [rabbit, Bethyl Laboratories (catalog no. IHC-0001) at 1:100], 8HG [rabbit, Novus Biologicals (catalog no. NB110-96878) at 1:100], NKX6.1 [rabbit, Cell Signaling Technology (catalog no. D8O4R) at 1:100 dilution], NKX2.2 [mouse, Developmental Studies Hybridoma Bank (catalog no. 74.5A5) at 1:40 dilution], PDX1 [goat at 1:10,000; (*75*)], NCL [rabbit, Cell Signaling Technology (catalog no. D4C7O) at 1:100], XBP1 [rabbit, Thermo Fisher Scientific (PA5-27650) at 1:100], HSPA5 [rabbit, Novus Biologicals (NBP1-06274) at 1:100], LC3A/B [rabbit, Cell Signaling Technology (catalog no. 12741) at 1:100], and LAMP1 [Developmental Studies Hybridoma Bank (catalog no. 1D4B) at 1:50]. The next day, the slides were rinsed in tris-buffered saline (TBS) 5× for 10 min each wash and incubated for 1 hour at room temperature with 4′,6-diamidino-2-phenylindole (DAPI) and secondary antibodies (1:400, Alexa Fluor 488, 555, 594, or 633). Last, samples were rinsed in TBS 4× for 10 min and mounted in VectaShield (Invitrogen) for confocal imaging. Microscopy of all slides from matched experiments was imaged in tandem.

### Confocal microscopy

Immunostained FFPE human pancreas slides were imaged using a Leica Microsystems Stellaris X5 confocal microscope fitted with a 20×/0.75 numerical aperture (NA) multi-immersion objective and a white-light laser. Three-dimensional (3D) confocal images were captured in line with a pixel size of ~270 nm in *XY* and 1 μm in *Z*, with a 15-μm stack size, and acquisition of fluorescence data was achieved using photon–arrival time gated photon-counting HyD2 detectors. The following fluorophore excitation (Ex) and emission (Em) bandwidths were used for data acquisition: DAPI: Ex: 405 nm, Em: 430 to 500 nm; Alexa Fluor 488: Ex: 499 nm, Em: 504 to 556 nm; Alexa Fluor 555: Ex: 553 nm, Em: 558 to 595 nm; Alexa Fluor 594: Ex: 590 nm, Em: 600 to 750 nm; and Alexa Fluor 633: Ex: 631 nm, Em: 640 to 750 nm.

### Confocal image analysis

Our approach to use time gated photon-counting detectors allowed us to determine how many short- and long-lived photons were generated for each pixel after excitation (“short” photon time bin 0 to 1 ns, “long” photon time bin 1.5 to 11.5 ns of pixel arrival times). Short-lived photons account for most of the sample autofluorescence [usually caused by leftover wax or lipofuscin bodies (*40*)], while long-lived photons yield a stronger and higher signal-to-noise ratio generated from each fluorophore used. Data from short-lived photons were placed into a separate 8-bit data channel to account for potential sample-to-sample and/or donor-to-donor variability in sample autofluorescence. After image acquisition, 3D confocal stacks were processed using ImageJ/FIJI, and maximum projection images from 8 to 12 optical sections were generated. Next, images were loaded onto QuPath for nuclei segmentation and cell border expansion, followed by quantification of absolute number of photons (herein referred to as intensity) detected in nuclear and/or cytoplasmic compartments. The DAPI channel was used as input for nuclei segmentation. Once the nuclei were segmented, we used a 3-μm border extension parameter to estimate the location of the cell cytosol. Last, the mean raw photon count values from each cell region and marker, i.e., short- and long-lived photon channels, were extracted and corrected to account for autofluorescence with the following formula: corrected mean intensity = (long-lived photon intensity values) − (short-lived photon intensity values). Next, we determined the mean intensity threshold for the insulin channel to identify beta cells versus non-beta cells. Only data from beta cells that were included in this classification were further analyzed and plotted. Beta cell size was quantified using ImageJ. Briefly, images were processed as maximum projections, as described above, and at least 10 beta cells per islet were segmented manually to extract beta cell area measurements. Analysis was performed in deidentified images to prevent any potential sample bias by the scientist performing the analysis.

### Confocal microscopy and image analysis of LC3-LAMP1 beta cells

Immunostained FFPE human pancreas slides were imaged using a Leica Microsystems Stellaris X5 confocal microscope fitted with a 63×/1.3 NA glycerol objective and a white-light laser. The 3D stacks (8 μm in size) were acquired in line and using Nyquist parameters predetermined via the “Lightning”-confocal mode. All images had an absolute pixel size of ~53 nm in X and Y and 0.3 μm in Z. After acquisition, confocal images were deconvolved in the LASX software using the adaptive mode. Before downstream analysis, all stacks were processed to generate maximum projections representing 4 μm of the original stack (maximum projection was centered around the stack’s equatorial section) and further denoised by applying a median filter of three pixels in X and Y (ImageJ). To analyze the size and density of LAMP1-positive granules in the beta cell cytoplasm, we created pixel classifiers [Aivia software (v10.5), Leica Microsystems] for INS and LAMP1 channels. This generated binary segmentation mask for insulin-positive or LAMP1-positive regions in each deconvolved image. A CellProfiler pipeline was created to quantify the area of LAMP1-positive granules occupying insulin-positive regions and their perimeter. Here, masked objects were created from Aivia-generated binary INS and LAMP1 masks. Next, the area occupied by INS- or LAMP1-positive pixels, as well as object shape descriptors were extracted to calculate LAMP1 area coverage and LAMP1-positive area perimeters. Colocalization of LC3 and LAMP1-positive pixels was calculated using Imaris software (v9.9, Oxford Instruments). Here, we used an automatic pixel threshold function to filter pixels in the LC3/LAMP1 channels for analysis, and the Mander’s coefficient representing the colocalization of LC3 and LAMP1 pixels was extracted. To analyze LC3-LAMP1 colocalization in beta cells only, we restricted the colocalization analysis using the binary mask created for the INS channel using Aivia software.

### Islet isolation and insulin secretion experiments

Islets were isolated at the ADI IsletCore as described in protocols deposited to the protocols.io public repository (*76*). Islets were handpicked to purity and cultured overnight at 37°C, 5% CO_2_ in Dulbecco’s modified Eagle’s medium supplemented with 10% fetal bovine serum and 1% penicillin-streptomycin. Insulin secretion was measured by static incubation at 37°C in KRB (115 mM NaCl, 5 mM KCl, 24 mM NaHCO_3_, 2.5 mM CaCl_2_, 1 mM MgCl_2_, 10 mM Hepes, and 0.1% BSA; pH 7.4) as outlined here (dx.doi.org/10.17504/protocols.io.wztff6n). Briefly, islets were preincubated for 2 hours in 1 mM glucose KRB. In triplicate, 15 islets per tube were subsequently incubated for 1 hour in KRB at 1 mM glucose, followed by 1 hour at 16.7 mM glucose, with supernatant collected after each hour. Acid/ethanol was used to extract insulin content, and samples were stored at −20°C until assayed for insulin (STELLUX, Alpco Diagnostics).

### Statistics

Statistical analysis of gene expression levels was performed within Seurat using default parameters. For IHC microscopy data, Student’s *t* test (Prism 9, GraphPad) was used to compare two groups, and a *P* value of <0.05 was considered significant. Data are shown as violin plots where median and upper and lower quartiles are displayed. For LC3-LAMP1 experiments, data are shown as scattered dot plots where each dot represents a single confocal image.

### Ethics statement

Written informed consent for use of pancreas in research was obtained from organ donors and/or their families by local organ procurement organizations. All donors were anonymized by the organ procurement organizations, and no identifying information was provided to or retained by the ADI IsletCore bio banking program. Usage of FFPE pancreas tissue was approved by the University of Alberta Human Research Ethics Board (Pro00013094) and Vanderbilt Institutional Review Board (no. 211203).

## Notes

View/request a protocol for this paper from *Bio-protocol*.
