# Supplementary material for: Aging compromises human islet beta cell function and identity by decreasing transcription factor activity and inducing ER stress
Source: Sci Adv. 2022 Oct 5;8(40):eabo3932. doi: 10.1126/sciadv.abo3932 (PMC9534504; doi:10.1126/sciadv.abo3932)
Supplement: Supplementary file 1 — Figs. S1 to S7 [file sciadv.abo3932_sm.pdf]

Science Advances
AAAS

Supplementary Materials for

Aging compromises human islet beta cell function and identity by decreasing transcription factor activity and inducing ER stress

Shristi Shrestha *et al.*

Corresponding author: Rafael Arrojo e Drigo, r.drigo@vanderbilt.edu

Sci. Adv. **8**, eabo3932 (2022)
DOI: 10.1126/sciadv.abo3932

The PDF file includes:

Figs. S1 to S7
Legends for tables S1 to S8

Other Supplementary Material for this manuscript includes the following:

Tables S1 to S8

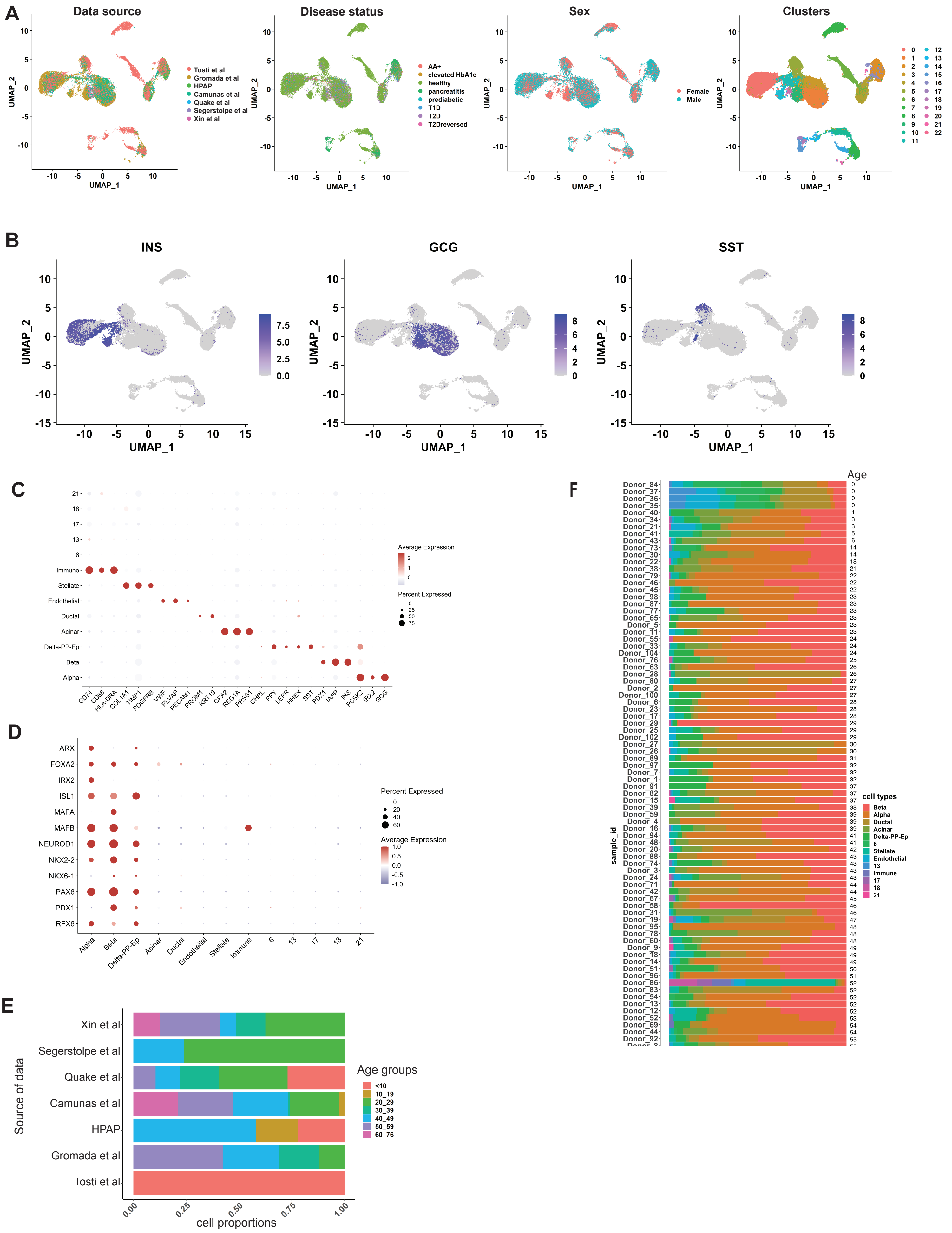

Supplementary Figure 1

Supplementary Figure 1 – Related to Figure 1. (A) UMAP coordinates as shown in Fig1, marked for each individual data source (i.e., study) used for meta-analysis, disease status, sex, and Seurat cluster. **(B)** Same as in (A), with the expression levels of INS, GCG or SST highlighted. **(C)** Dot plot indicating the relative expression levels and percentage of cells in identified cell-type clusters (rows) expressing cell-type marker genes (columns). **(D)** Same as in (C), however this panel highlights endocrine cell-enriched genes. **(E)** Stacked bar graph showing the fraction of cells from each data source (rows) broken down by each decade of age included in our analysis. **(F)** Stacked bar graph showing the fraction of each cell type identified per donor included in our meta-analysis. Donor identifiers are shown on the left and the graph is ranked in an ascending order based on donor age (right labels).

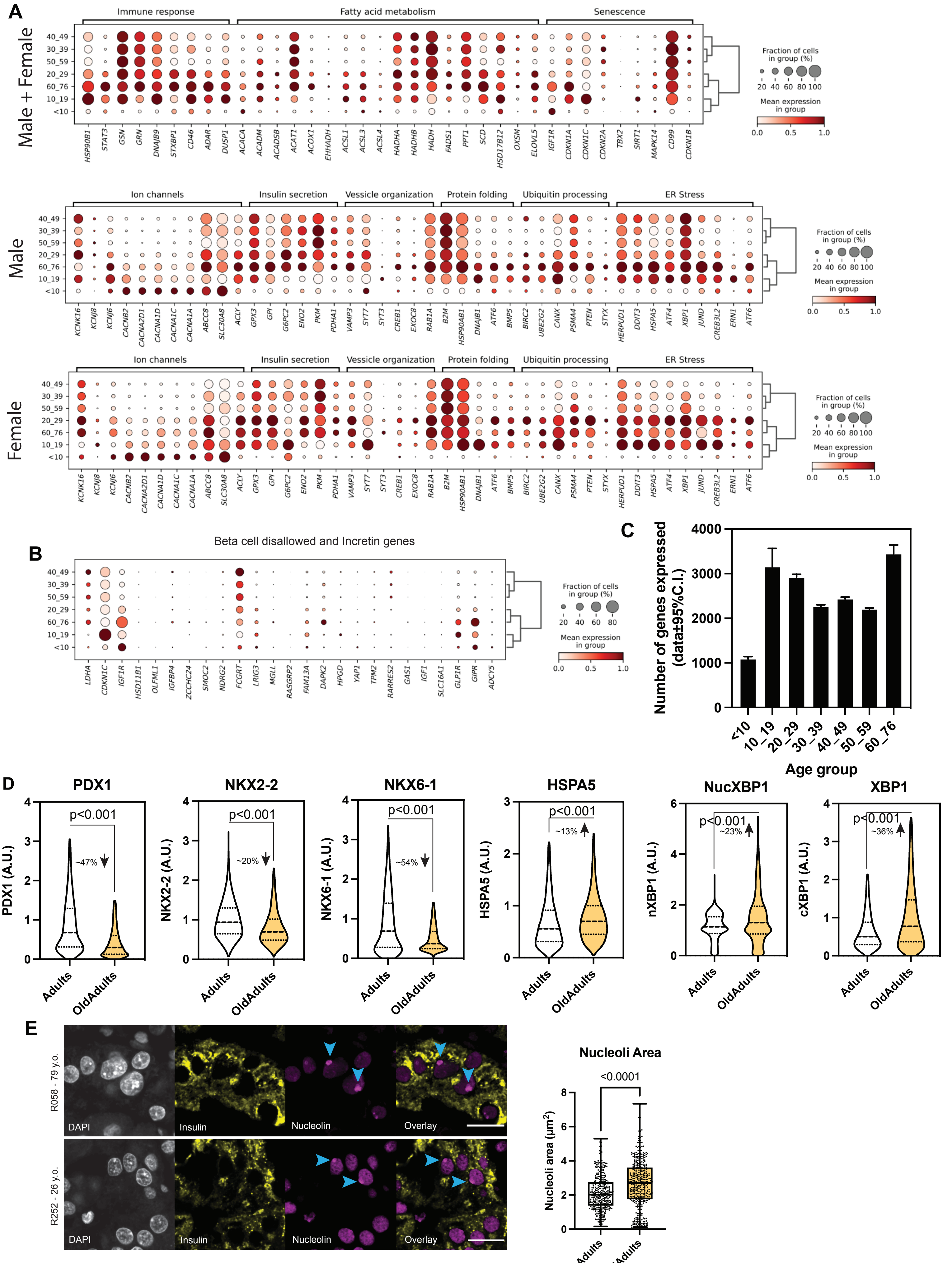

Supplementary Figure 2

Supplementary Figure 2 – Related to Figure 2. (A) Dot plot with HCA highlighting the expression levels of genes associated with immune response, fatty acid metabolism, and senescence. **(B)** Dot plot with HCA highlighting the expression levels of beta cell disallowed, aging and incretin genes. **(C)** Number of genes expressed in beta cells from donors at different decades of age. **(D)** Quantification of mean fluorescence intensity for PDX1, NKX6-1, NKX2-2, HSPA5 and XBP1 (nuclear (nuc) and cytosolic compartments). **(E)** Representative maximum projection images of human islets stained with Insulin and Nucleolin. Blue arrowheads indicate the location of the beta cell nucleolar compartment; quantification of nucleolar size is shown in the box plot on the right. Each dot represents an individual beta cell nucleus. In (D), data from n=6 adult and n=6 old adult donors. Number of beta cells quantified by age group for each marker: PDX1: 12,032 adult and 22,119 old adult cells; NKX6.1 and NKX2.2: 18,304 adult and 18,575 old adult cells; HSPA5: 17,474 adult and 25,469 old adult cells; XBP1: 26,382 adult and 48,623 old adult beta cells. In (E), data from n=5 adult and n=6 old adult donors, with 431 adult and 419 old adult cells analyzed.

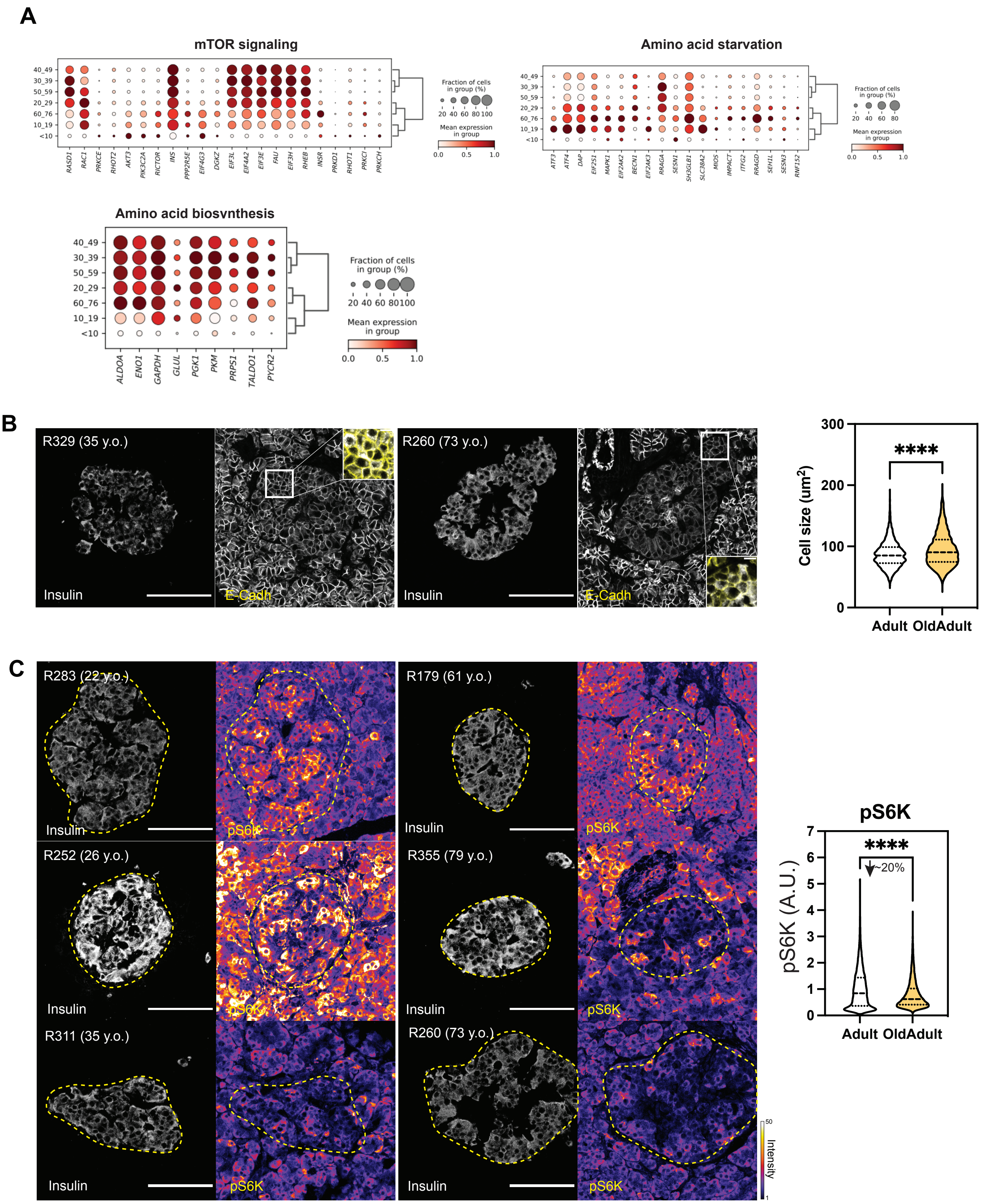

Supplementary Figure 3

Supplementary Figure 3 – Related to Figure 2. (A) Dot plots with HCA highlighting the expression levels of genes associated with mTOR signaling, amino acid starvation and biosynthesis pathways. **(B-C)** Immunohistochemistry and confocal microscopy of human pancreas FFPE samples from adults and old adults. Human islets were stained with (B) Insulin and E-cadherin, or (C) with Insulin and phospho-S6 kinase (pS6K) antibodies. Quantification of beta cell size or cytosolic pS6K levels are shown as violin plots on the right side of (B-C), respectively. In (B), data from n=6 adult and n=6 old adult donors, with 2,798 adult and 3,481 old adult beta cells analyzed. In (C), data from n=6 adult and n=6 old adult donors, with 9,453 adult and 19,708 old adult beta cells analyzed.

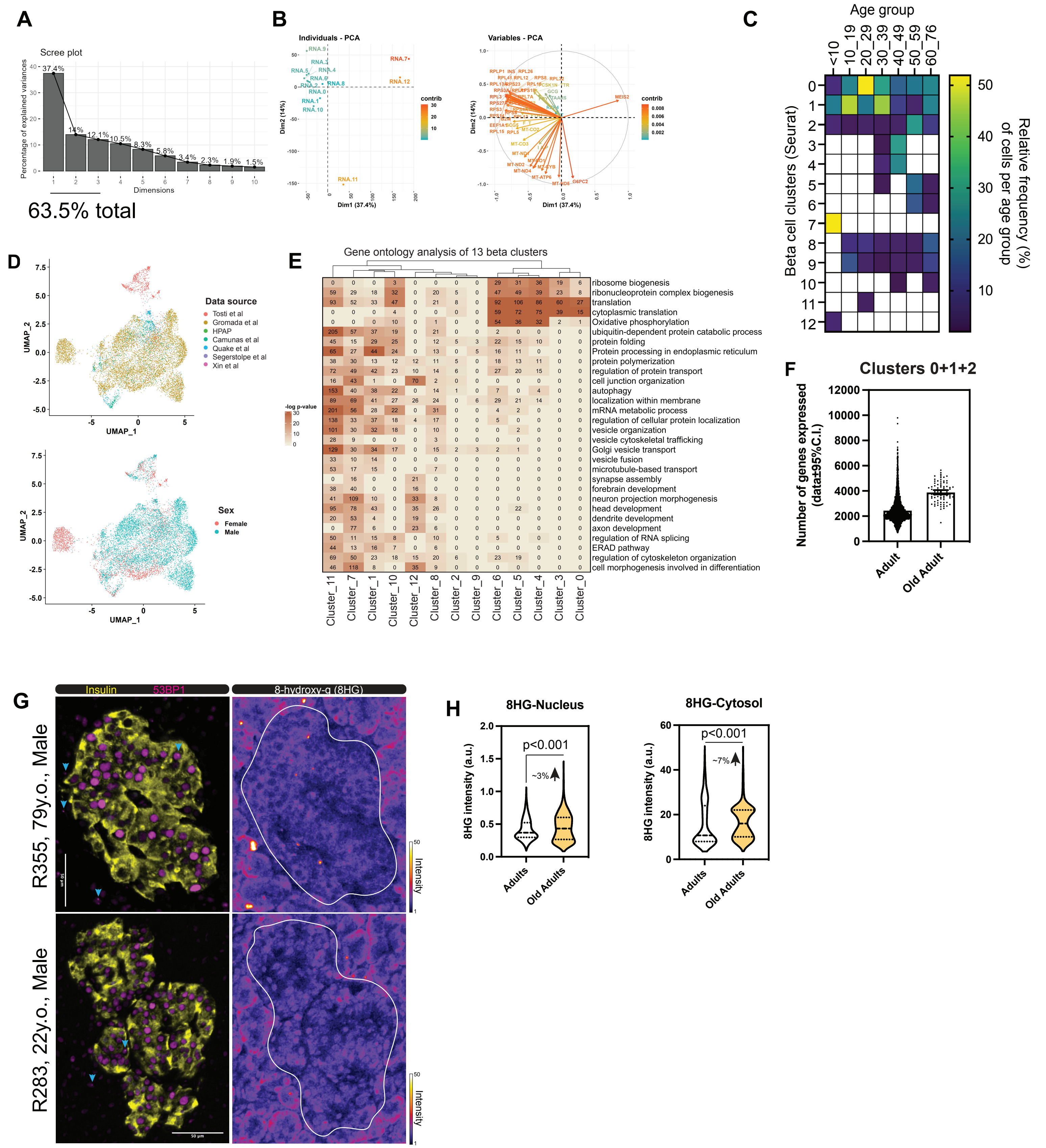

Supplementary Figure 4

Supplementary Figure 4 – Related to Figure 3. (A) Scree plot showing the most relevant dimensions that drive changes in gene expression in our meta-analysis. **(B)** Left, graph of individual PCA dimensions 1 and 2. Note how most beta cell clusters (i.e., RNA”x”) cluster together while rare beta cell clusters (#11, 12) or younger (#7) beta cells are significantly different in these dimensions. Right, graph of variables identifying of gene identity and correlation with overall gene expression pattern. The further away vectors are from a PC origin, more influence on that PC a given gene has; the vectorial direction is correlative to PC and angles between variables represent the following: 90° no correlation, 180° anti-correlation, 0° correlation. **(C)** Relative distribution of beta cell Seurat clusters as a function of donor age categorized by age decades. **(D)** UMAP as shown in Figure 2A-B overlayed with identity of the original data set and sex for each annotated cell. **(E)** Pathway enrichment analysis of genes represented in each identified beta cell cluster from Seurat. **(F)** Number of genes expressed in beta cells from adult and old adult donors in the most abundant Seurat clusters (0-to-2). **(G)** Representative maximum projection images of human islets stained with Insulin, 53BP1 and 8HG. Blue arrowheads indicate the location of 53BP1-positive nuclear foci. **(H)** Quantification of 8HG mean intensity in the nucleus or cytosolic compartments of beta cells from adult and old adults. In (H), data from n=3 adult and n=3 old adult donors, with 5,430 adult and 8,445 old adult beta cells analyzed.

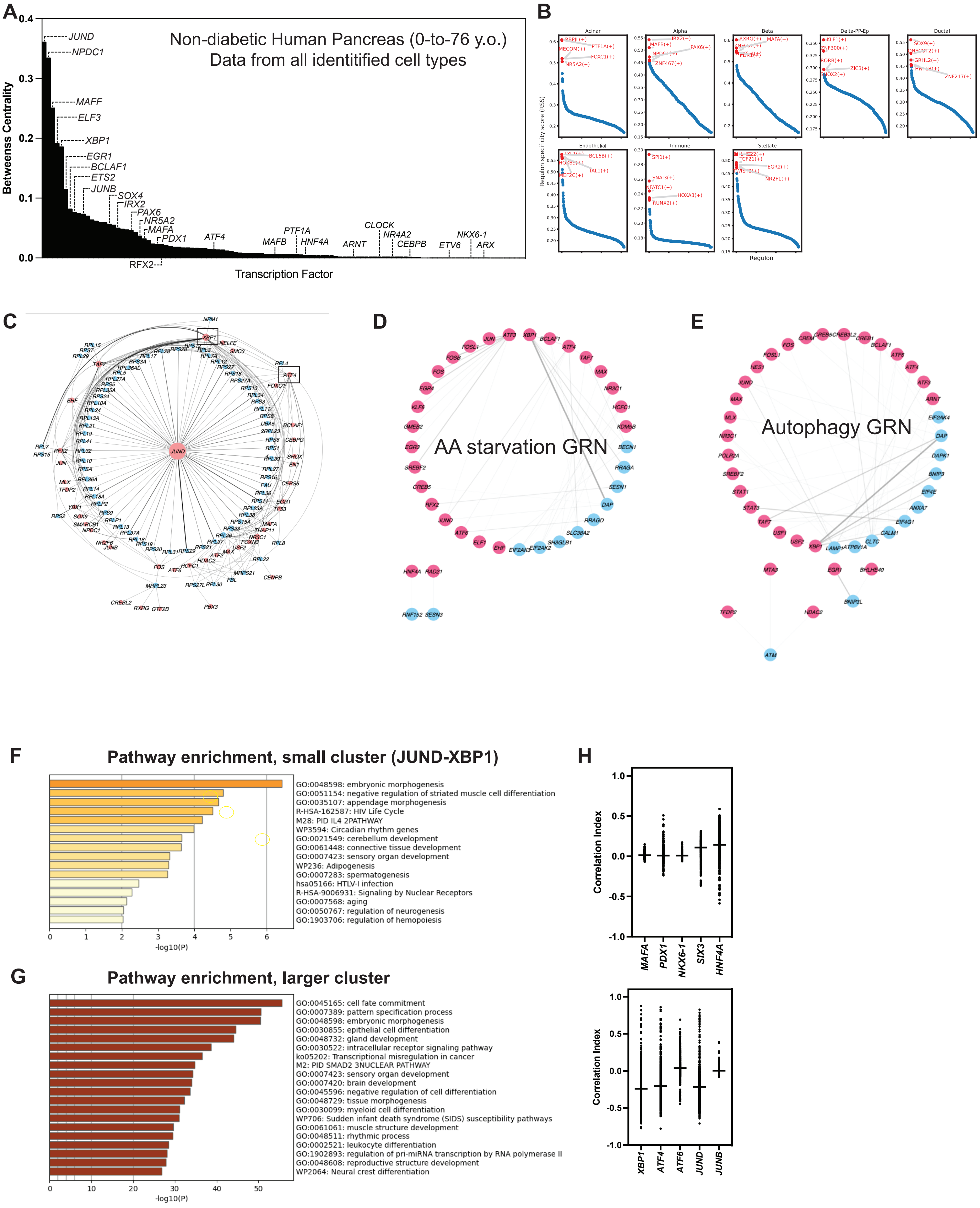

Supplementary Figure 5

Supplementary Figure 5 – Related to Figure 4. (A) Histogram of the "Betweenness Centrality" index for each TF node identified using pySCENIC in the human pancreas. This index quantifies the relative importance of each node within the network. Identity of major transcription factors identified in different cell types are shown in relationship to each other. **(B)** Regulon scores for major cell types of the human pancreas analyzed with SCENIC. Higher the score, higher the confidence regarding the identification of TF-gene pairs. Top 5 regulons for each cell types are identified and shown in red. **(C-F)** GRN plots of JUND and ATF4 regulons, and of TFs associated with AA starvation and autophagy pathways. Black outline bounding boxes highlight the location of ER stress TFs XBP1 and ATF4 in the JUND regulon. **(G)** and **(H)**, pathway enrichment analysis of TF clusters identified using Pearson's correlation matrix shown in Fig4. **(I)** Aligned dot plot with the Pearson correlation index of TFs linked to beta cells (top) or ER stress response (bottom).

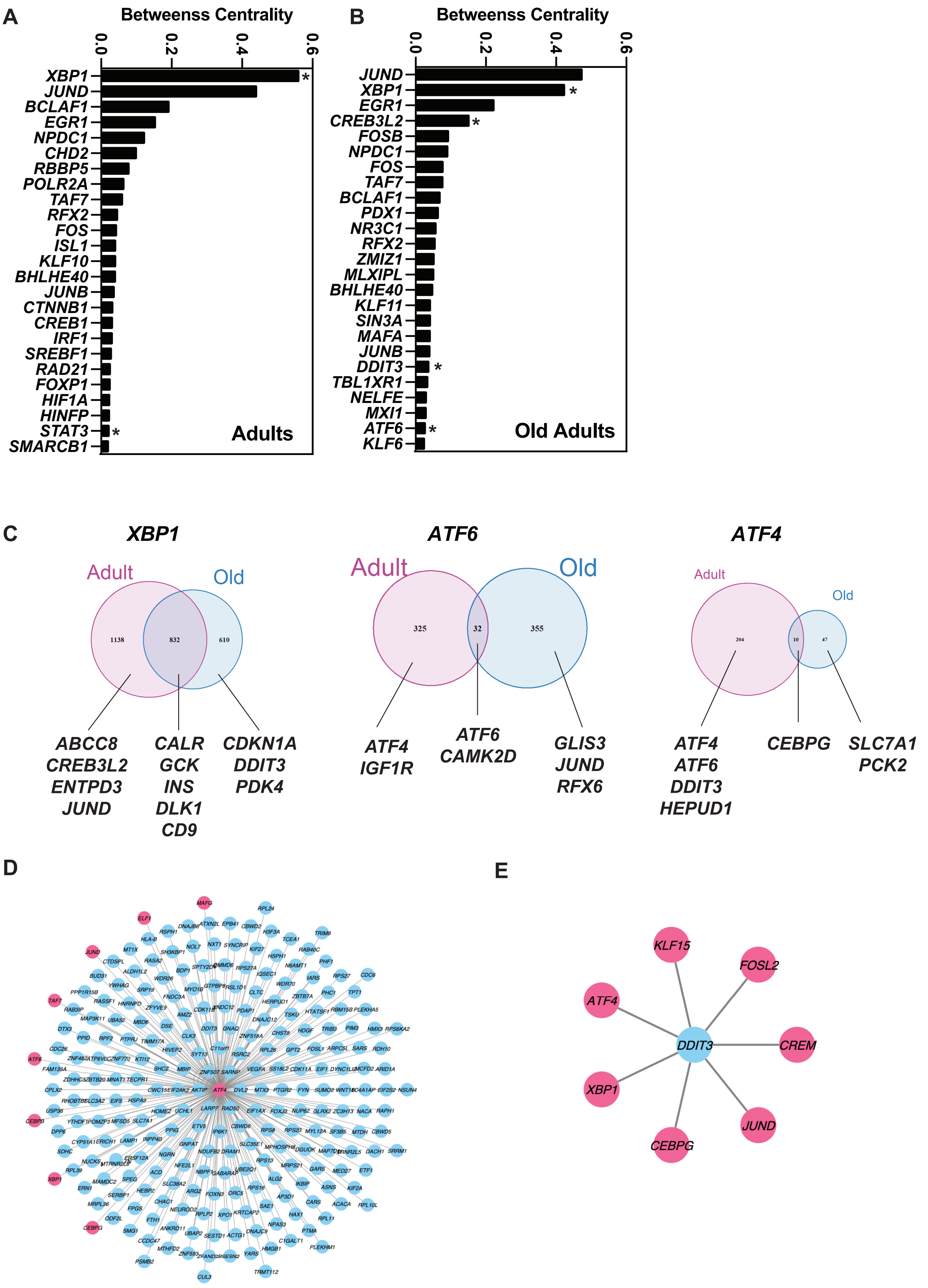

Supplementary Figure 6

Supplementary Figure 6 – Related to Figure 5. (A-B) Histogram of the "Betweenness Centrality" index for the top 25 TF nodes identified using pySCENIC in human beta cells from adults and old adults. Asterisks indicate TFs related to stress-response pathways **(C)** Venn diagrams of unique or shared genes targeted by XBP1, ATF6 and ATF4 in adult and old adult beta cells. Example of genes in each category is listed at the bottom of each graph. **(D-E)** ATF4 and DDIT3 GRNs in human beta cells.

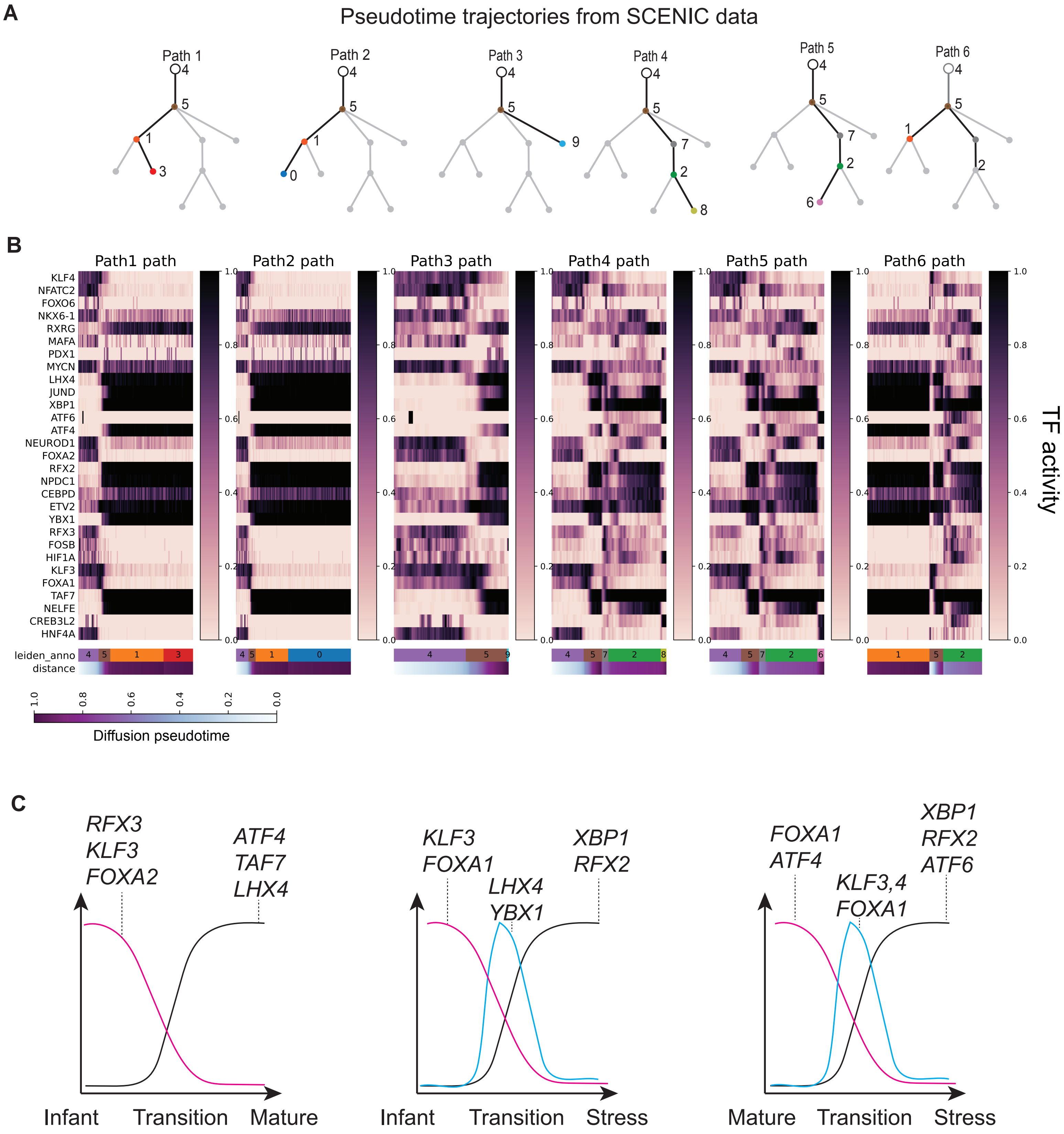

Supplementary Figure 7

Supplementary Figure 7 – Related to Figure 6. (A) PAGA pseudotime tree highlighting the six different beta cell trajectories. **(B)** Heatmaps illustrating the binary activity of select TFs across six different trajectories. TFs are organized in rows and pseudotime trajectories are arranged in columns. Pseudotime regions occupied by individual Leiden clusters are shown at the bottom of each heatmap. **(C)** Graphical summary of the data from (B), highlighting select TFs with distinct activity patterns in beta cells in the pseudotime tree.

Table S1: Single cell RNAseq dataset metadata.

Table S2: Differentially expressed genes in Beta cells vs all other pancreatic cell types based on the non-parametric Wilcoxon rank sum test, and genes used for classification of cell types in the human pancreas.

Table S3. (A) Donor Metadata for GSIS studies, including RRIDs (when available). **(B)** Mean islet insulin content and descriptive statistics in each age group analyzed in our GSIS studies.

Table S4: List of beta cell TF regulons (n=609) identified using pySCENIC.

Table S5: List of regulons in healthy beta in adult age group (20-59yrs) cells. IM = "im portance metrics" that associates the strength of TF-target relation.

Table S6: Overlap of GRNs between ATF4, ATF6 and XBP1 in N.D. beta cells from adults, old adults, and infants

Table S7: Donor metadata for samples used in confocal microscopy experiments

Table S8: List of Software Algorithms and R packages used to analyze data.
